# In Search of the Ninth Discipline: The History of Pathophysiology, with an Emphasis on Pathophysiology in Varna, Bulgaria—Celebrating 100 Years of Pathophysiology in Bulgaria

**DOI:** 10.7759/cureus.2404

**Published:** 2018-04-02

**Authors:** George S Stoyanov, Galina Naskovska, Emran Lyutfi, Rumiana Kirneva, Kameliya Bratoeva

**Affiliations:** 1 Department of General and Clinical Pathology, Forensic Medicine and Deontology, Medical University – Varna "Prof. Dr. Paraskev Stoyanov", Varna, BGR; 2 Department of Physiology and Pathophysiology, Division of Pathophysiology, Faculty of Medicine, Medical University – Varna "Prof. Dr. Paraskev Stoyanov", Varna, BGR; 3 Student, Faculty of Medicine, Medical University – Varna "Prof. Dr. Paraskev Stoyanov", Varna, BGR; 4 Department of Physiology and Pathophysiology, Division of Pathophysiology, Medical University – Varna "Prof. Dr. Paraskev Stoyanov", Varna, BGR

**Keywords:** pathophysiology, pathological physiology, history of medicine, pathophysiology in bulgaria, pathophysiology in varna

## Abstract

Pathophysiology is a medical science whose subject is the change in regulatory mechanisms related to the onset, development, and outcome of diseases. The first lectures on pathophysiology were held in 1790 at the University of Erfurt, Germany, by Professor Augustus Hecker, who in 1791 also published the first work on the discipline – "Grundriss der Physiologia pathologica" in 770 pages. The teaching of pathophysiology as an independent discipline was introduced by academician Viktor Pashutin at the University of Kazan, Russia in 1874. Academician Pashutin called this new discipline “Pathological Physiology and Experimental Medicine.” Despite the persuasiveness of Pashutin that pathological anatomy and pathophysiology are inseparable parts of a whole, his students, academician Nikolay Anichkov and Prof. Semyon Khalatov, implemented the so-called “divorce” due to the different, though complementary, approaches and methodologies of the two ideological fields. By Royal Decree on November 29, 1917, in the Bulgarian State Gazette, amendments were published in the law on the national education, which introduced new university “disciplines and departments”. Under number nine in the law is the discipline of “Pathological Physiology and Experimental Medicine”. Due to various factors, the Pathological Physiology and Experimental Medicine department was the only one of the first 25 departments not to be established. The beginning of the training for pathophysiology in Bulgaria was laid by Prof. Vassil Mollov and Assoc. Prof. Minko Dobrev, however due to their untimely deaths, the course lasted only three years (1936–1939) and was not continued in the next academic year. At the beginning of the academic year 1946/47, two assistants in pathophysiology were enrolled in the Department of Pathological Anatomy at Sofia University. The following year a separate department was formed at the newly founded Plovdiv University and shortly after at Sofia University. For the 100 years since its legislative establishment, 82 years since its unofficial start and 71 years since its academic establishment pathophysiology in Bulgaria has distinguished itself by scientific, administrative and clinical contributions. In its 57 years in Varna, Bulgaria pathophysiology has widely carried out that tradition with immense contributions.

## Introduction and background

Pathophysiology is a medical science whose subject is the change in regulatory mechanisms related to the onset, development, and outcome of diseases. The first lectures on pathophysiology were held in 1790 at the University of Erfurt, Germany, by Professor Augustus Hecker (born 1.07.1763 – died 11.10.1811), who in 1791 also published the first work on the discipline – “Grundriss der Physiologia pathologica” in 770 pages [1]. In the 1830s, a distinguished school started forming, with members such as Johannes Müller (born 14.07.1801 – died 28.04.1985), Hermann von Helmholtz (born 31.08.1821 – died 8.09.1894), the father of cellular theory Rudolph Virchow (born 13.10. 1821 – died 5.09.1902) and the founder of experimental medicine Julius Friedrich Cohnheim (born 20.07.1839 – died 15.08.1884).

The teaching of pathophysiology as an independent discipline was introduced by Academician Viktor Pashutin (born 28.01.1845 – died 2.02.1901) at the University of Kazan, Russia in 1874. As head of the Department of Pathology, he decided to introduce a new discipline relying on the German pathophysiology society. Academician Pashutin called this new discipline “Pathological Physiology and Experimental Medicine”.

Despite the persuasiveness of Academician Pashutin that pathological anatomy and pathophysiology are inseparable parts of a whole, his students Academician Nikolay Anichkov (born 1885 – died 7.12.1964) and Professor Semyon Khalatov (born 26.02.1884 – died 17.05.1951), authors of the first model of atherosclerosis in the world, implemented the so-called “divorce” due to the different, though complementary, approaches and methodologies of the two ideological fields.

## Review

On the 10^th^ of April 1918, the dream of a number of Bulgarian activists, dating from before the liberation, was fulfilled for the establishment of a Medical Faculty on the territory of Bulgaria at Sofia University. Although the result of attempts to do so for nearly two decades, the structural and legislative bodies were not prepared for such a step, with a severe lack of staff, legal regulations and curriculum preparation [2].

**Figure 1:**
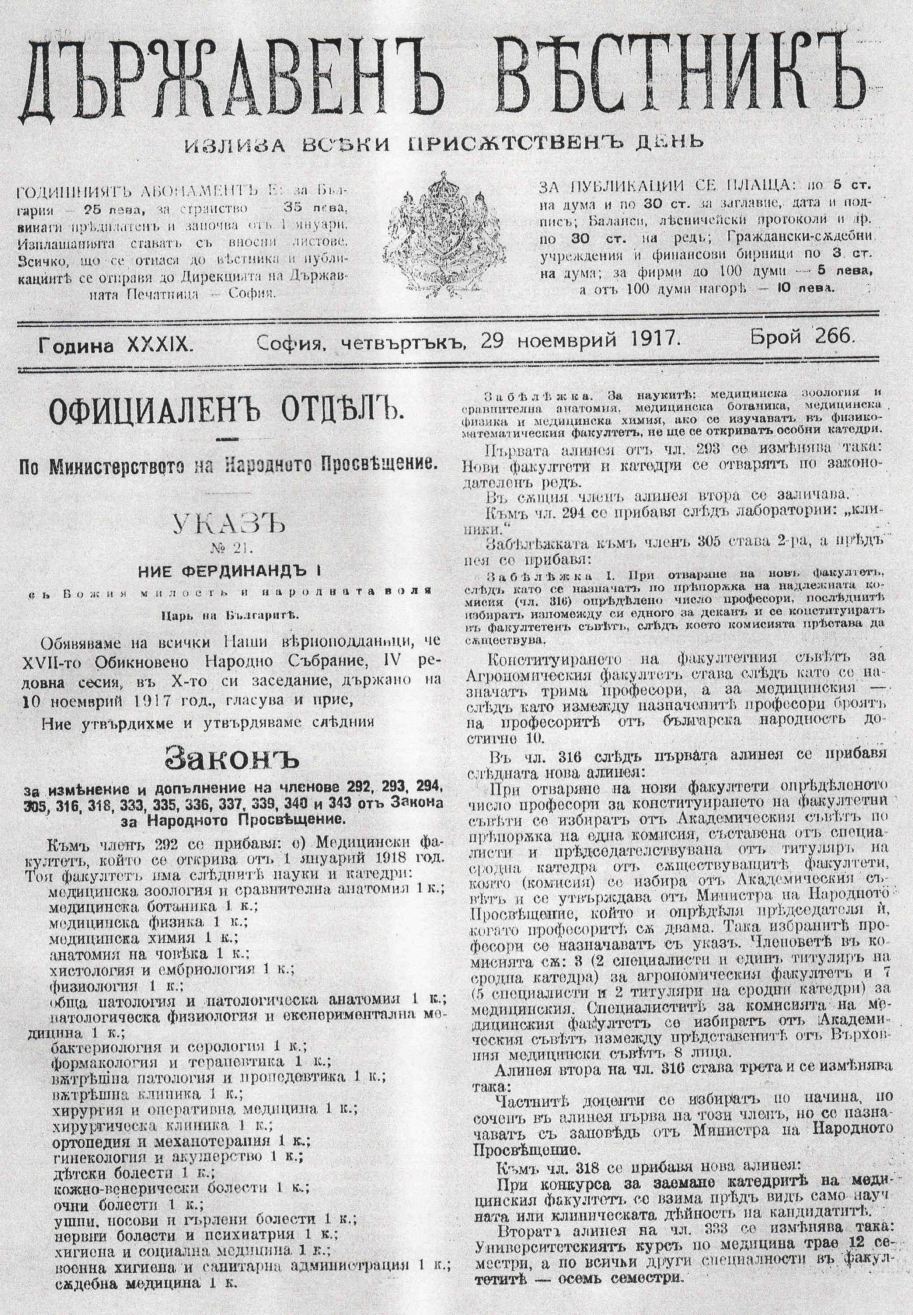
State Gazette (in Bulgarian). Year XXXIX, issue 226.

By Royal Decree on November 29, 1917, in the State Gazette (year XXXIX, issue 226), amendments were published in the law on the national education, which for the first time introduces new university “disciplines and departments” (Figure 1) [2].

These were to be the basic areas of study, established as a result of previous experience with the Sofia Feldsher School and the German and Russian medical tradition, which were with the greatest political influence at that time, on the background of the dominant French medical tradition, implemented by practicing physicians at that time.

Under number nine in the law is the discipline of “Pathological Physiology and Experimental Medicine”.

Due to various factors playing a role in driving the faculty, the staff set, and the necessary equipment, this was the only one of the first 25 departments not to be established, despite being included in the semester program of the third-year students. Even in the first year of the faculty, this presented as a serious problem, due to the transfer of two third-year students from foreign universities.

As a result of the many organizational problems, a pathophysiology course was not established. The basics of the discipline were reallocated between General Pathology, Propaedeutic of Internal Diseases and Propaedeutic of Surgical Diseases. The first few courses took the so-called Selected Chapters of Physiology in their last year of study, where the pathophysiological mechanisms of a number of disease processes were largely interpreted [2].

The beginning of the training in pathophysiology was laid by the Professor of Internal Medicine Vassil Mollov (born 22.11.1875 – died 22.12.1938) at Sofia University, who in 1936–1937 delivered a cycle of lectures on clinical pathophysiology. The lecture course was later taken up by Associate Professor Minko Dobrev (born 5.06.1898 – died 14.03.1939), who prepared a full course of 32 lectures. This gives reason to accept Associate Professor Dobrev as the first Bulgarian pathophysiologist. Due to the death of Professor Mollov and not long after Associate Professor Dobrev, the course lasted only three years (1936–1939) and was not continued in the next academic year [2].

The first officially recognized Department of Pathophysiology in Bulgaria was formed in 1947 at the newly founded Plovdiv University, under the supervision of Professor Luben Telcharov (born 17.11.1907 – died 1995), who specialized in Grenoble, Berlin, and Würzburg.

At the beginning of the academic year 1946/47, two assistants in pathophysiology were enrolled in the Department of Pathological Anatomy at Sofia University. The following year a separate department was formed under the supervision of the graduate and specialist in the Union of Soviet Socialist Republics (USSR) Associate Professor Stefan Ivanov Pisarev (born 28.02.1901 – died 12.03.1983) [2,3].

The newly founded Department of Pathophysiology found a difficult beginning of its existence. The methodology of teaching was rooted in Russian methods, while the majority of the medical community in Bulgaria was now already educated by the German model. At the same time, the newly formed Department faced well-established university disciplines with decades of experience, which have left a scientific footprint and have already produced their own professors.

Despite these problems, the department had a rapid pace of development. The experimental practices were adopted and approved by Soviet scholars, such as Professor Andrei Dimitrijevich Ado (born 12.01.1909 – died 29.10.1997).

The Department had its first participation in international scientific forums in 1948, 1950 and 1951. For the first four years of its existence in the newest department of the Medical Faculty at Sofia University and later the reformed Higher Medical Institute (HMI), Sofia, five Philosophy doctorate (Ph.D.) theses were successfully defended and two more were assigned. For the same period of time, from all 39 departments, only five Ph.D. students successfully defended Ph.D.s [2].

Over the same period over 50 full-text publications were published, seven of which in journals in the USSR, German Democratic Republic, France, England and Romania [2].

In 1958, a unique goal was set, which has not yet been achieved on a worldwide scale. The Department of Pathological Physiology required its transfer to a clinical setting on the territory of the Internal Diseases Clinic and a change of name to Department of Clinical Pathophysiology. The purpose of the Department's restructuring was to carry out complex clinical and experimental activities on its territory, together with clinicians and pharmacologists [2].

Little data remains for most of the first Bulgarian Pathophysiologists.

The first pathophysiologists at Plovdiv University

N. Gaydadzhieva, M. Todorova, and Y. Pantev were the first assistants enrolled with the establishment of the Department of Pathophysiology at Plovdiv University. Sadly, today, no accessible biographical data has survived for them.

The first pathophysiologists at Sofia University and the reformed HMI-Sofia

V. Serafimov Dimitrov – no biographical data, Ph.D. thesis “Importance of Some Disturbances of the Central Nervous System for the Pathogenesis of Allogenic Diabetes” [2].

D. Kiprov – no biographical data, Ph.D. thesis “Pathomorphological studies on the dynamics of experimental arthritis and myocarditis” [2].

R. I. Sabeva – no biographical data, died prior to the defense of her Ph.D. thesis on cardiovascular disease [2].

Liebknecht Argirov Dimitrov (born 14.06.1922 – date of death unknown). Graduated from HMI Sofia in 1951. Ph.D. thesis “Influence of the nervous system on the emission of 17-ketosteroids in the urine”. Main interests in the field of adrenal gland pathophysiology, radiobiology, and hematology. Habilitated as a Professor in 1974 [2,3].

Maria Petrova Papazova (born 3.06.1925 – date of death unknown). Graduated from HMI Sofia in 1950. Ph.D. thesis “Experimental data on the influence of the nervous system on stomach functions in inflammation of the intestine”. One of the few Bulgarian medics at that time to specialize in the United States of America (USA) – Urbana (1966–1967). Main scientific interests in the pathophysiology of digestion. Habilitated as a Professor in 1973 and a major figure in the early years of the Institute of Experimental Medicine with two recognized inventions [2,3].

Ivan Alexandrov Popdimitrov (born 2.07.1923 – date of death unknown). Graduated from HMI Sofia in 1950. Ph.D. thesis “Role of the central nervous system in the pathogenesis of tuberculosis”. Main scientific interests in the field of protein plasma substitutions, parenteral nutrition, pathophysiology of protein metabolism and organ hemodynamics. Habilitated as a Professor in 1971. Head of the Department of Pathophysiology and Experimental Medicine at the Bulgarian Academy of Sciences, 1958–1971. Head of the Department of Pathological Physiology at HMI Varna, 1971–1988. Deputy Dean of the Faculty of Medicine at HMI Varna, 1973–1977. Habilitated as a Professor in 1971 with three inventions and three rationalizations [2-4].

**Figure 2:**
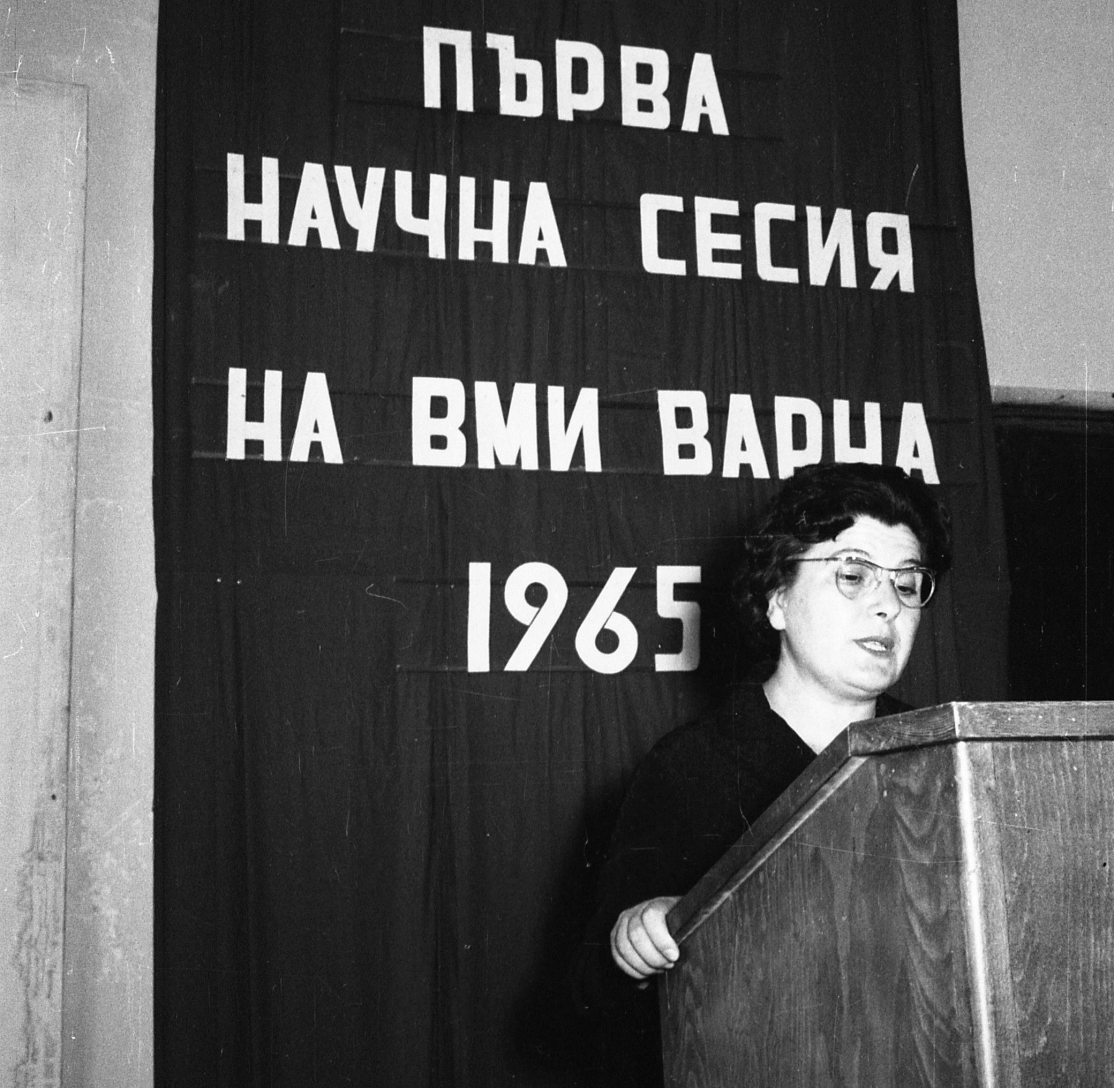
Professor Zdravka Kemileva, 1961. The photograph was taken during the opening ceremony of the First Scientific Meeting of HMI Varna. Background writing in Bulgarian translates to First Scientific Meeting of HMI Varna 1965. HMI: Higher Medical Institute

Zdravka Savova Kemileva (born 26.11.1919 – died 2002) (Figure 2). Graduated from Sofia University in 1944. Ph.D. thesis “Role of the neuroses on the course of experimental arthritis”. Founder and First Head of the Department of Pathophysiology at HMI Varna, 1961–1971. Third Rector of HMI Varna (1.04.1963–1.07.1966). Head of the Department of Pathophysiology at Institute for Specialization and Improvement of Physicians (ISIP), 1970–1981, and a unified Department of Pathophysiology in ISIP and HMI Sofia, 1981–1984. Major scientific interests in thrombosis pathophysiology, metabolism, and cellular type inflammatory reactions. Member of the Editorial Board of the Journal of Experimental Morphology, from its establishment and later Editor-in-Chief. Habilitated as a Professor in 1966 [2-4].

Pathophysiology in Varna, Bulgaria

Two of the forefathers of Bulgarian Pathophysiology moved to Varna with the establishment of HMI Varna – Professor Kemileva and Professor Popdimitrov.

Professor Kemileva and Professor Popdimitrov left their mark not only on the teaching process, scientific and experimental activities in the early years of the newly formed University, but also gave significant administrative contributions, serving as Rector and Deputy Dean of the Faculty of Medicine, respectively [4].

Under the supervision of Professor Kemileva, a number of changes were introduced in the university's research activities. The scientific periodical was renamed from Annual scientific papers – HMI Varna to Scripta Scientifica Medica, with the official language being changed from Bulgarian and Russian to English, 1965. The first scientific meeting of the newly formed institute was also held in 1965 [4].

The mandate of Professor Kemileva saw the establishment of new departments: Ear, Nose and Throat Diseases; Internal Diseases and Therapy; Ophthalmology; Social medicine; Forensic Medicine, as well as the establishment of a Central Diagnostic Laboratory of Virology and Microbiology [4].

Anelia Dyakova Uzunova (born 15.05.1933). Graduated from HMI Sofia in 1957. Ph.D. thesis “Experimental myocarditis and arthritis against a background of altered immunological reactivity”, 1964 – the first successfully defended dissertation at HMI Varna. One of the few Bulgarian medics at that time to specialize in the USA – Washington, 1970–1972 and 1974–1977. Later appointed as Head of the Department of Pathological Physiology at HMI Pleven, 1982–1999. Main scientific interests in the field of thymus involvement in immunological processes, pathogenesis of thrombosis and influence of eicosanoids in arterial thrombosis. Habilitated as a Professor in 1990 [4].

Kolyo Angelov Velikov (born 22.10.1927). Graduated from HMI Sofia in 1958. Ph.D. thesis “Experimental myocarditis and arthritis in adult rat dimeters”, 1972. Main scientific interests in the field of rheumocardiology. After his retirement in 1988 until his death, he worked as a district and general physician in the village of Hursovo, Shumen. Habilitated as an Associate Professor in 1974 [4].

**Figure 3:**
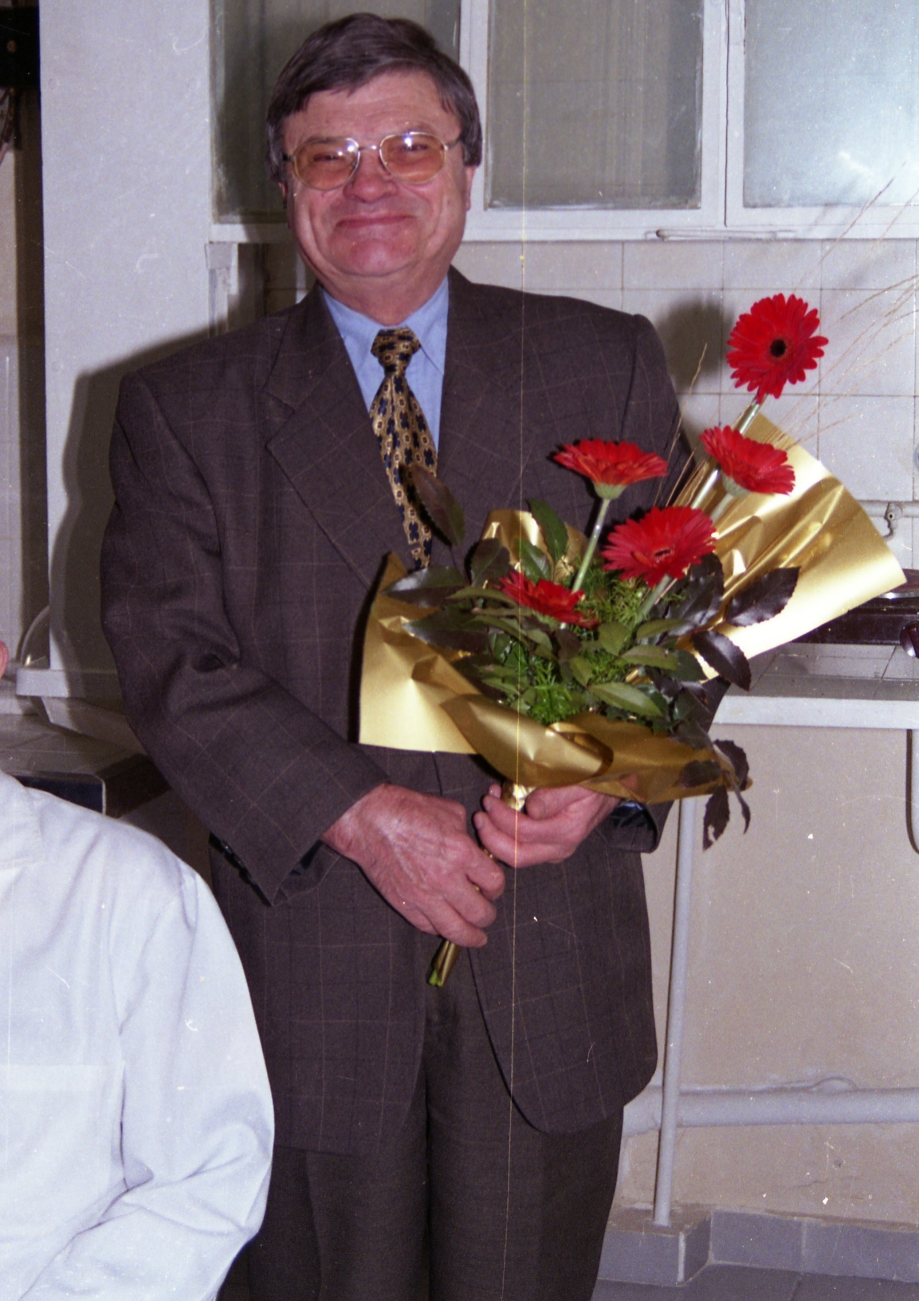
Associate Professor Ivan Kozarev on his retirement, 2003.

Ivan Kolev Kozarev (born 20.01.1937) (Figure 3). Graduated from HMI Sofia 1962. Ph.D. thesis “Influence of the Thymus on the immune-allergic reactivity of the organism”, 1973. Main scientific interests in the field of immuno-allergic processes and thermal injuries. Founder and First Head of the Department of Pathophysiology at the affiliate of HMI Varna in Dobrich, 1978–1988. Subsequent Head of the Department of Pathophysiology at HMI, 1988–2000. His Ph.D. students include Ganka Bekyarova, MD and Radko Radev, MD, who served terms as Head of the department after his retirement. His guidance saw the habilitation as Associate Professor of his Ph.D. student Ganka Bekyarova. Habilitated as an Associate Professor in 1978 [4].

**Figure 4:**
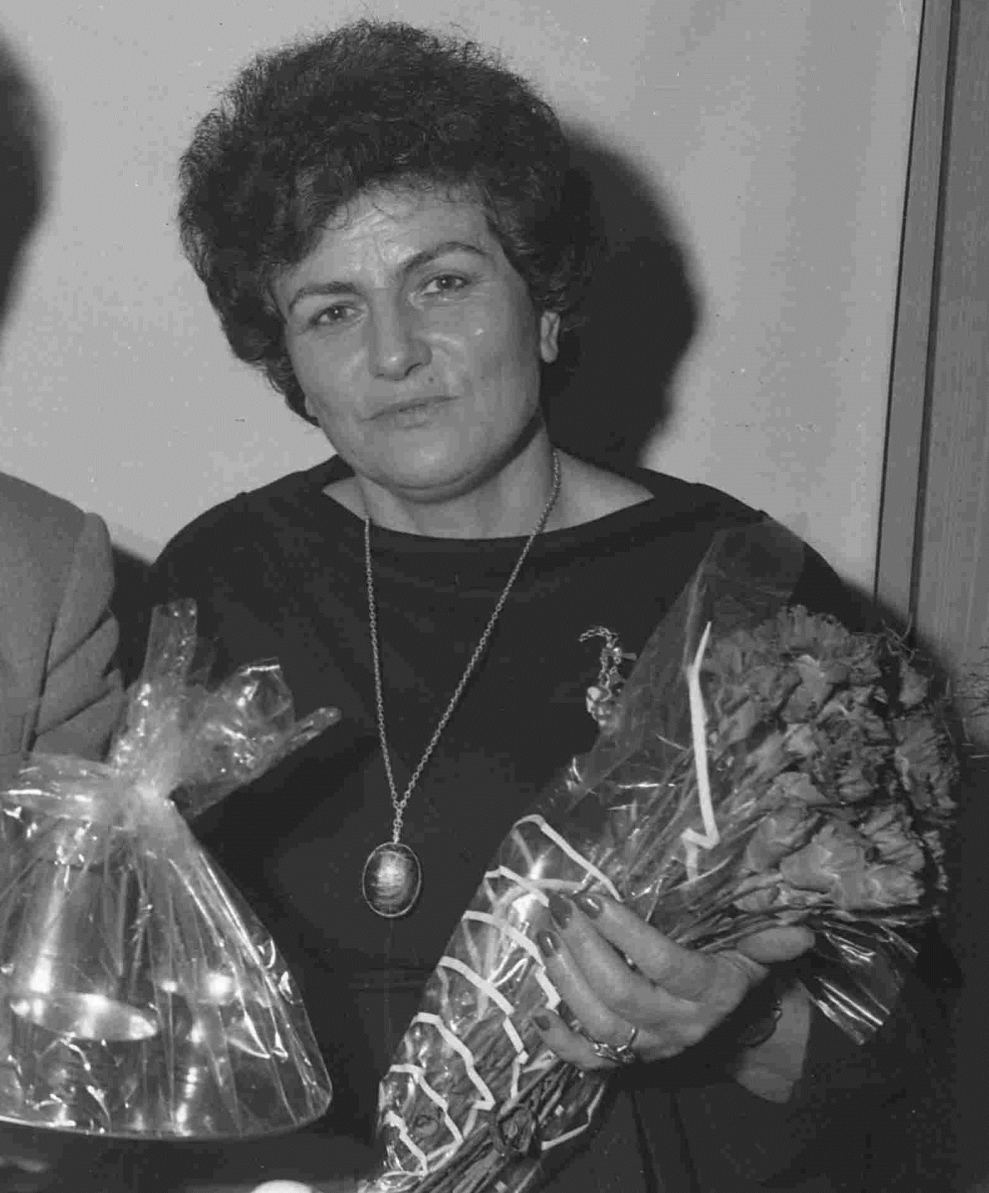
Associate Professor Kalinka Demireva, circa 1985.

Kalinka Marinova Mirchera-Demireva (born 6.03.1936) (Figure 4). Graduate from HMI Sofia in 1960. Ph.D. thesis “Studies on pathogenesis and prophylaxis of experimental atherosclerosis”, 1977. Main scientific interests in the field of atherosclerosis, transplantable tumor strains, gerontology, and geriatrics. Head of the experimental radioisotope laboratory at the Department of Pathophysiology. Member of the Department of Pathophysiology at the Faculty of Medicine of the Thracian University – Stara Zagora from 1999 until her retirement. Habilitated as an Associate Professor in 1991 [4].

**Figure 5:**
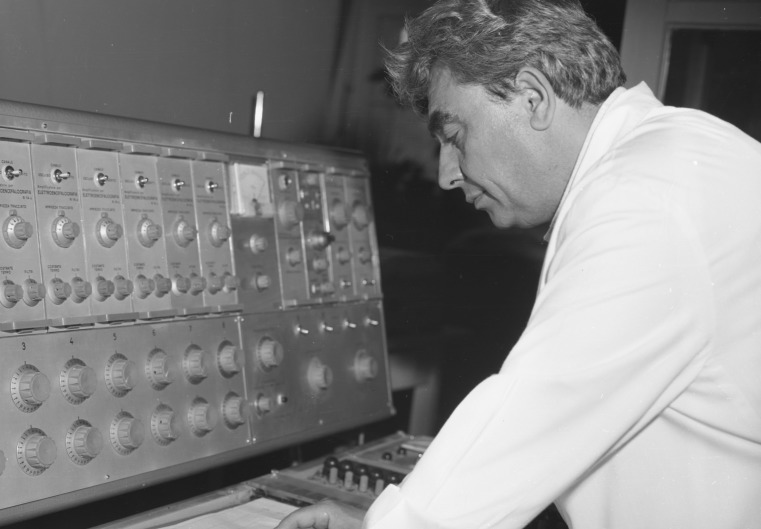
Associate Professor Tsvetan Tsekov calibrating an EEG apparatus, circa 1980. EEG: Electroencephalogram

Tsvetan Toshev Tsekov (born 9.12.1931) (Figure 5). Graduated from HMI Sofia in 1955. Ph.D. thesis “Relational time dependence and spinal changes of preliminary preparation for arbitrary movement”, 1975. Main scientific interests in the field of regulation of action potential motor activity and electrophysiological organ activity. Associate, and subsequently Head of the Central Experimental Electrophysiological Laboratory at the Department of Pathophysiology, HMI Varna. Head of the Central Research and Research Laboratory at HMI Varna, 1979. Habilitated as an Associate Professor in 1979 [4].

## Conclusions

For the 100 years since its legislative establishment, 82 years since its unofficial start and 71 years since its academic establishment pathophysiology in Bulgaria has distinguished itself by scientific, administrative and clinical contributions. In its 57 years in Varna, Bulgaria pathophysiology has widely carried out that tradition with immense contributions. The Department has cast a Rector, Deputy Dean of the Faculty of Medicine and Head of the Central Scientific Research Laboratory. For that time period three professors, seven associate professors, and more than 20 Ph.D. graduates have successfully defended their theses. The Department in Varna, Bulgaria has also hosted an Experimental Radioisotope Laboratory and a Central Experimental Electrophysiology Laboratory, now defunct, where electromyograms and electroencephalograms, on patients from the Clinics of Neurology, Neurosurgery, and Psychiatry, were carried out as a diagnostic medium.
